# Microencapsulated Pomegranate Modifies the Composition and Function of High-Density Lipoproteins (HDL) in New Zealand Rabbits

**DOI:** 10.3390/molecules25143297

**Published:** 2020-07-21

**Authors:** Alan Dorantes-Morales, Diego Estrada-Luna, Rocío Bautista-Pérez, Gabriel Betanzos-Cabrera, María Luna-Luna, Cristóbal Flores-Castillo, Gilberto Vargas-Alarcón, José Manuel Fragoso, Óscar Pérez-Méndez, Elizabeth Carreón-Torres

**Affiliations:** 1 Department of Molecular Biology, Instituto Nacional de Cardiología “Ignacio Chávez”, Juan Badiano 1, Sección XVI, Tlalpan, Mexico City 14080, Mexico; alandm10@outlook.com (A.D.-M.); diego.estrada.luna@gmail.com (D.E.-L.); rociobtst@yahoo.com (R.B.-P.); mjluna.qfb@gmail.com (M.L.-L.); flockmx@gmail.com (C.F.-C.); gvargas63@yahoo.com (G.V.-A.); mfragoso1275@yahoo.com.mx (J.M.F.); 2 Área Académica de Nutrición y Toxicología Clínica, Instituto de Ciencias de la Salud, Universidad Autónoma del Estado de Hidalgo, Ex-Hacienda de la Concepción, Tilcuautla, Pachuca de Soto 42080, Hidalgo, Mexico; gbetanzo@uaeh.edu.mx; 3 School of Engineering and Sciences, Tecnológico de Monterrey, Campus Ciudad de México, Calle del Puente #222, Ejidos de Huipulco, Tlalpan C.P. 14380, México City, Mexico

**Keywords:** high-density lipoprotein, pomegranate microencapsulated, supplementation, profile lipid, cardioprotective function

## Abstract

Previous studies demonstrated that pomegranate, which is a source of several bioactive molecules, induces modifications of high-density lipoproteins (HDL) lipid composition and functionality. However, it remains unclear whether the beneficial effects of pomegranate are related to improvement in the lipid components of HDL. Therefore, in this placebo-controlled study, we characterized the size and lipid composition of HDL subclasses and assessed the functionality of these lipoproteins after 30 days of supplementation with a pomegranate microencapsulated (MiPo) in New Zealand white rabbits. We observed a significant decrease in plasma cholesterol, triglycerides, and non−HDL sphingomyelin, as well as increases in HDL cholesterol and HDL phospholipids after supplementation with MiPo. Concomitantly, the triglycerides of the five HDL subclasses isolated by electrophoresis significantly decreased, whereas phospholipids, cholesterol, and sphingomyelin of HDL subclasses, as well as the HDL size distribution remained unchanged. Of particular interest, the triglycerides content of HDL, estimated by the triglycerides-to-phospholipids ratio, decreased significantly after MiPo supplementation. The modification on the lipid content after the supplementation was associated with an increased resistance of HDL to oxidation as determined by the conjugated dienes formation catalyzed by Cu^2+^. Accordingly, paraoxonase-1 (PON1) activity determined with phenylacetate as substrate increased after MiPo. The effect of HDL on endothelial function was analyzed by the response to increasing doses of acetylcholine of aorta rings co-incubated with the lipoproteins in an isolated organ bath. The HDL from rabbits that received placebo partially inhibited the endothelium-dependent vasodilation. In contrast, the negative effect of HDL on endothelial function was reverted by MiPo supplementation. These results show that the beneficial effects of pomegranate are mediated at least in part by improving the functionality of HDL, probably via the reduction of the content of triglycerides in these lipoproteins.

## 1. Introduction

The inverse correlation between high-density lipoproteins (HDL)-cholesterol and coronary heart disease is well known and a role of these lipoproteins against atherosclerosis has been suggested [1,2,3,4]. However, the mechanisms and components of HDL that explain the anti-atherogenic functionality of these lipoproteins remain unclear.

HDLs are macromolecular complexes that include bioactive molecules within their structure, other than lipids and proteins. The components of HDL vary according to diverse pathophysiological conditions and result in functional differences of HDL; the amount of paraoxonase-1 (PON1), an enzyme carried in plasma by HDL that confers most of their antioxidant properties to these lipoproteins [5], and the anti-inflammatory capacity of HDL depend on HDL structure [6,7,8]. In this context, recent studies have demonstrated that HDL are internalized to the cytoplasm of cultured cells and deliver cholesterol and sphingomyelin during this process [9,10,11]. By delivering lipids to the endothelial cells, HDL attenuated the expression of the intracellular adhesion molecule−1 (ICAM−1) and favored the phosphorylation of endothelial nitric oxide synthase (eNOS) [11]. The regulation of endothelial cells by HDL depends upon the lipid content of these lipoproteins. Therefore, HDL likely drive lipids from the gut and from the liver not only to the endothelial cell, but also to other peripheral cells to maintain the balance of lipids in their membranes. Consequently, the functionality of the lipid rafts and the activity the membrane proteins, among others, are guaranteed [12,13]. During lipid delivery to the cells, HDL may also supply other molecules, including microRNAs (miRNAs) [14], acute phase proteins [15], and numerous hydrophobic compounds that may be associated with HDL structure [16].

Therefore, physiological changes in the lipid content of HDL [11,17,18], particularly during the postprandial hypertriglyceridemia [18] may impact the biological function of these lipoproteins in the cell. Currently, HDL quality and functionality seem to be the most important target for developing new or alternative therapies, including the use of functional foods. In this context, pomegranate is a source of flavonoids and phenolic compounds, among others, with hypolipemiant and antioxidant potential; in vivo and in vitro studies demonstrated that pomegranate alters HDL lipid composition and functionality [19,20]. Similarly, a previous study from our group demonstrated that after supplementation with microencapsulated pomegranate (MiPo) in women with acute coronary syndrome (ACS), postprandial triglyceridemia significantly decreased [21]. In that study, isolated HDL partially inhibited the endothelial-dependent vasodilation in vitro, and such effect was exacerbated during the postprandial state. Interestingly, the harmful effects of HDL on endothelial function were reverted after supplementation with MiPo and a concomitant increase of PON1 activity [21]. However, it remains unclear whether the pomegranate exerts an indirect beneficial effect on HDL through the improvement of the postprandial triglyceridemia or a direct effect that enhances HDL functionality. In order to explore whether pomegranate directly improves the HDL functionality or not, the aim of the present study was to characterize the size and lipid composition of HDL subclasses and to evaluate the antioxidant properties of HDL, including the resistance to oxidation and PON1 activity. We further examined the effect of HDL on endothelial-dependent vasodilation in vitro after a daily supplementation of MiPo. Since the elevated levels of fasting or postprandial plasma triglycerides are a confounding factor for our objectives, the study could not be conducted in humans. Therefore, we performed this study on New Zealand white rabbits, species that is characterized by mild triglycerides increases during the postprandial state, thus providing an animal model suitable for reaching controlled conditions for the aim of our study [22].

## 2. Results

### 2.1. Biochemical Characteristic and HDL Lipids Profile

The biochemical data of the experimental groups are presented in Table 1. After 30 days of supplementation with MiPo, total cholesterol and triglycerides significantly decreased 20.8% and 27.0%, respectively. In contrast, the placebo group that received the encapsulating agent of MiPo, maltodextrin (Section 4), showed a slight but significant increase of 13.3% in the triglyceride’s plasma concentrations after intervention. Moreover, supplementation with MiPo was associated with a 26.5% reduction in HDL-triglycerides (HDL-Tg) plasma levels and increases of 15.3% and 7.3% for HDL-cholesterol (HDL-C) and HDL-phospholipids (HDL-Pho), respectively. In the placebo group, we observed a 10.5% decrease in HDL-C after intervention (Table 1).

**Figure 1:**
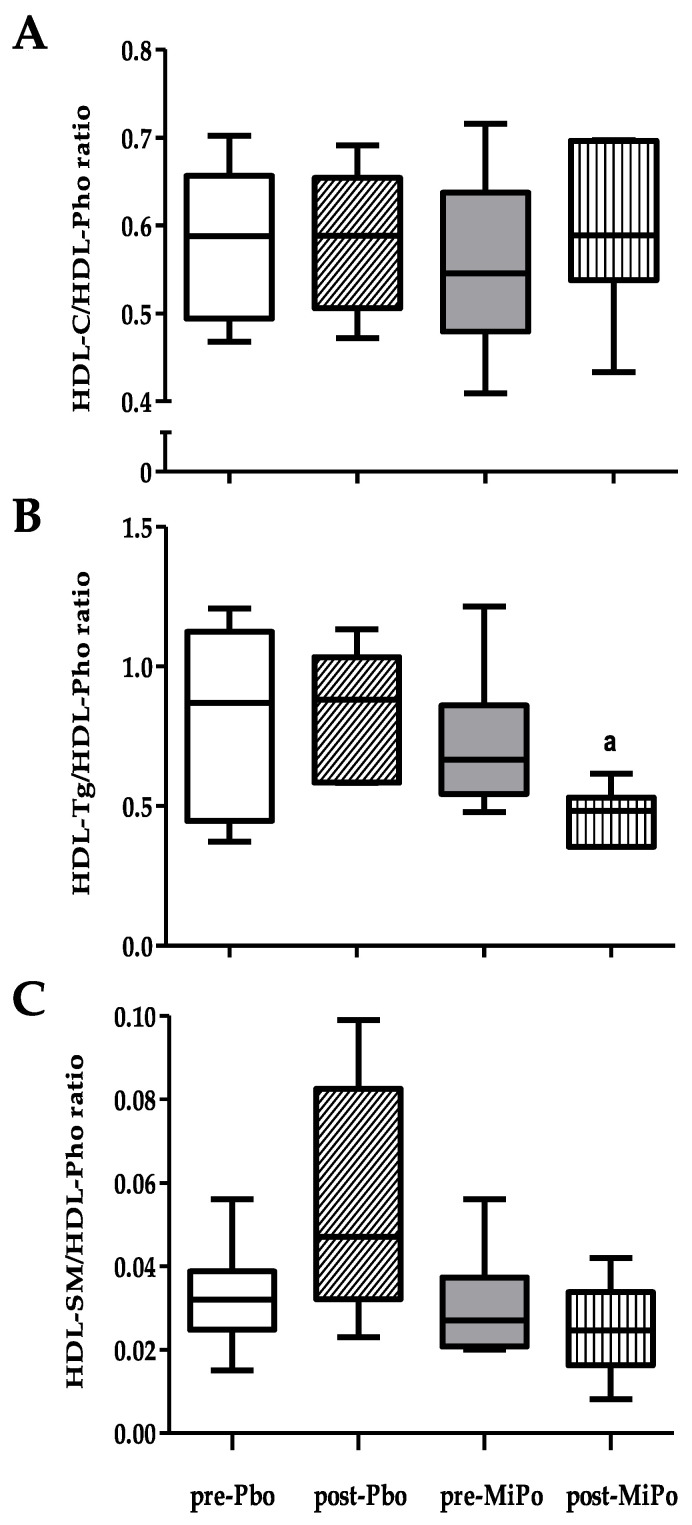
Lipid plasma concentration ratios of HDL. The ratios were calculated in both MiPo and placebo groups, before (pre-) and after (post-) of 30 days of supplementation with MiPo or administration with maltodextrin as placebo (Pbo). (**A**) HDL-cholesterol-to-phospholipids ratio. (**B**) HDL-triglycerides-to-phospholipids ratio. (**C**) HDL-sphingomyelin-to-phospholipids ratio. Data are expressed as median (horizontal lines) and interquartile range (boxes). Mann–Whitney U test. ^a^ *p* < 0.05 Baseline (pre-) vs. after 30 days of supplementation with MiPo (post-).

We further determined the HDL-Tg/HDL-Pho, HDL-C/HDL-Pho, and HDL-sphingomyelin (HDL-SM)/HDL-Pho ratios as markers of HDL lipid composition [23]. Our results showed a significant 27.3% decrease in the HDL-Tg/HDL-Pho ratio after intervention with MiPo; in the placebo group, we observed no significant changes (Figure 1).

### 2.2. Size and Lipid Composition of HDL Subclasses

The size distribution of HDL subclasses remained unchanged after supplementation with MiPo (Table S1). In contrast, the triglycerides of the HDL2a, HDL3b, HDL3a, and HDL3c subclasses decreased on average 34% after of supplementation with MiPo. Concerning phospholipids and sphingomyelin concentrations of HDL subclasses, we did not find significant changes in any of the groups (Table 2).

### 2.3. Kinetics of Conjugated Dienes Formation in HDL (Oxidation)

**Figure 2:**
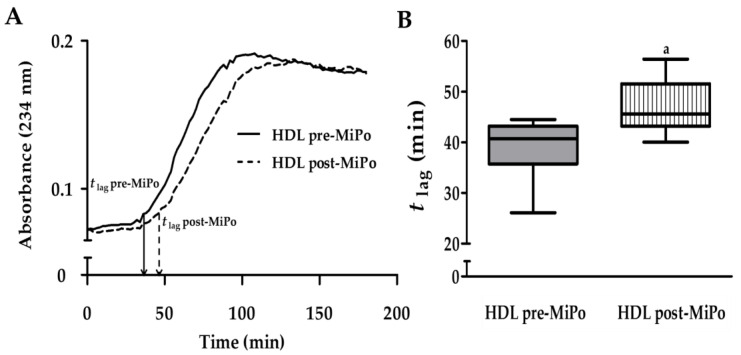
Effect of MiPo on the resistance to oxidation of HDL. The conjugated dienes formation catalyzed by Cu^2+^ was determined within HDL from rabbits, before (pre-MiPo) and after (post-MiPo) supplementation with microencapsulated pomegranate. (**A**) Kinetics of conjugated dienes formation determined by spectrophotometry at 234 nm; the curves represent the calculated mean of the individual kinetics pre-MiPo (solid line, *n = 6*) and post-MiPo (dashed line, *n = 6*), normalized to the same initial value. (**B**) Calculated lag time (*t*_lag_); horizontal lines and boxes represent the median and interquartile range, respectively. Mann–Whitney U test, ^a^ *p* < 0.05, pre- vs. post-MiPo.

The kinetics of conjugated dienes formation catalyzed by copper ion (Cu^2+^) was examined spectrophotometrically as described in Methods. The lag time (*t*_lag_) was considered as the time from the addition of the Cu^2+^ until the start of the log phase of dienes formation; the longer the *t*_lag_ the higher the resistance to generation of conjugated lipid hydroperoxides of the HDL. The mean of the individual kinetics before and after MiPo supplementation are shown in Figure 2A. The curve of dienes formation within HDL was shifted to the right after MiPo supplementation; accordingly, the *t*_lag_ was 22.5% longer after MiPo (Figure 2B), indicating an increased resistance of HDL to oxidation provided by pomegranate.

### 2.4. Paraoxonase-1 (PON1) Activity

**Figure 3:**
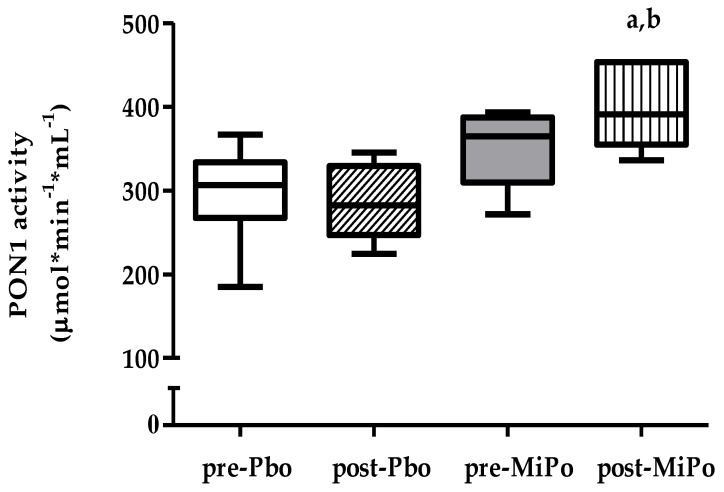
Effect of MiPo on paraoxonase-1 (PON1) activity. PON1 activity was determined in both MiPo and placebo groups, before (pre-) and after (post-) 30 days of supplementation with MiPo or administration with the encapsulating agent of MiPo, maltodextrin (Pbo). Data are expressed as median (horizontal lines) and interquartile range (boxes). Mann–Whitney U test. ^a^ *p* < 0.05, pre- vs. post-MiPo; ^b^ *p* < 0.05, placebo vs. MiPo after 30 days.

To explore the possibility that the changes in the structure of HDL particles had an impact on the functionality of these particles, we measured the activity of PON1. The results showed that MiPo supplementation induced a significant increase of 13.5% in PON1 activity (Figure 3).

### 2.5. Effect of HDL on Endothelial Function

**Figure 4:**
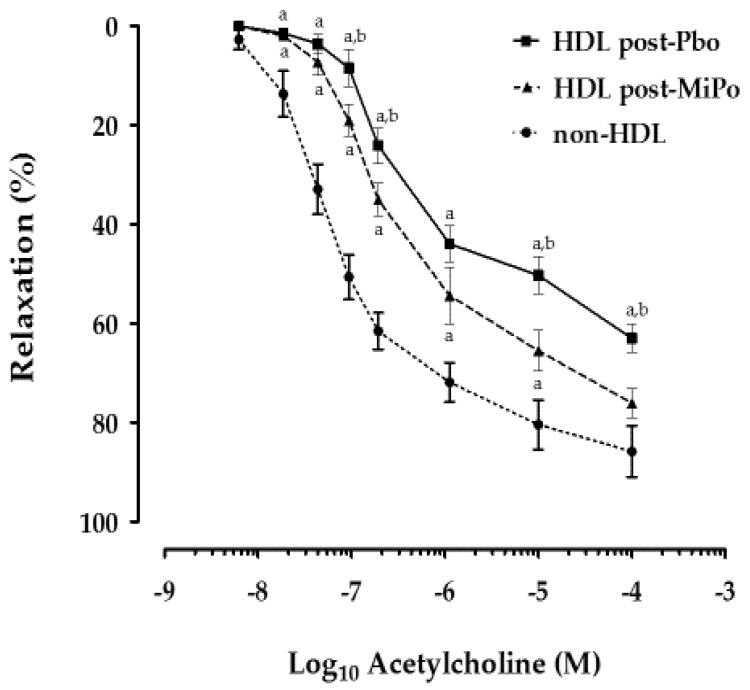
Effect of MiPo on endothelial-dependent relaxation of rabbit aortic rings incubated with HDL from rabbits. Curves represent the percentage of relaxation of pre-contracted aorta rings with phenylephrine as a function of the logarithm of increasing acetylcholine concentration. Aorta rings were incubated with HDL from rabbits after 30 days of supplementation with MiPo (post-MiPo, black triangles) and administration with the encapsulating agent of MiPo, maltodextrin (post-Pbo; black squares). Each point represents the mean and standard error of the mean (SEM; *n* = 6). As reference, a curve of aorta rings without HDL is included (non-HDL, black circles). ANOVA, Least significant difference (LSD) post-hoc test, ^a^ *p* < 0.05 vs. control, ^b^ *p* < 0.05 vs. MiPo after 30 days of supplementation.

We determined the endothelial-dependent vasodilation of rabbit aorta rings as a marker of functionality in the presence of HDL. Our results showed that the vasodilation of aorta rings incubated with HDL isolated from the plasma of rabbits that received placebo or MiPo supplementation was significantly lower compared with the vasodilation of aorta rings incubated in the absence of HDL. This effect was observed along the whole range of concentrations of acetylcholine (10^–8^–10^–4^ M). However, the vasodilation of the aorta rings in the presence of isolated HDL after supplementation with MiPo was significantly higher compared with the placebo group in the same range of concentrations of acetylcholine (Figure 4).

## 3. Discussion

In the present study, MiPo supplementation induced changes in the composition and functionality of HDL particles in male New Zealand White rabbits. We observed an improved lipid profile after 30 days of supplementation with MiPo: significant decreases in total cholesterol (CT), triglycerides (Tg), and non-HDL-sphingomyelin (non−HDL-SM) and increases in HDL-cholesterol (HDL-C) and HDL-phospholipids (HDL-Pho). Rabbits increased about 40% their corporal weight during the study; considering that the maltodextrin used as placebo is inert, the triglycerides increase, and the reduction of HDL-C were associated to the natural growth and aging of the animals. Concomitantly, the plasma concentrations of HDL-triglycerides (HDL-Tg) and the triglycerides of the subclasses of HDL decreased significantly. These changes could be related to an improvement in the HDL functionality, i.e., increased PON1 activity, increased resistance for formation to Cu^2+^-induced conjugated lipid hydroperoxides, and the lack of an inhibitory effect on vasodilation dependent on the endothelium after supplementation with MiPo. These results agree with those reported in women with acute coronary syndrome after MiPo consumption [21]; however, in the present study we demonstrated the HDL modifications in an animal model characterized by very mild postprandial increases of plasma triglycerides, indicating that pomegranate has a beneficial effect on HDL functionality, independently of an improvement of the postprandial triglyceridemia. Moreover, the modifications of the lipid profile after MiPo in this study were similar that those observed in human. This fact represents a confirmation of the beneficial effects of pomegranate and suggests that the results of the present study could be similar in humans [21].

The role of sphingomyelin (SM) is still controversial; SM may be a risk factor for CAD [24]; low-density lipoproteins (LDL) are the main carriers of SMs and ceramides that have been associated with an increased risk of coronary heart disease [25]. Moreover, SM decreased in the 5 subclasses of HDL in patients with metabolic syndrome after weight loss [26]. However, the presence of SM in reconstituted HDL is necessary to regulate ICAM-1 and to induce eNOS phosphorylation [11]. Therefore, the impact of SM seems to be related to the carrying lipoprotein [25]. In this study, the decrease of non-HDL-SM with no changes of HDL-SM may reflect an additional potential benefit of pomegranate as nutraceutical product. In addition to SM, other characteristics of HDL such as the proportion of unsaturated acyl chains, and particularly the lysophosphatidylcholine that affect HDL function [27] deserve to be analyzed in future studies.

The beneficial effects of pomegranate have been attributed to its high content of bioactive compounds, and principally to the polyphenols such as ellagic and gallic acids, anthocyanins, and ellagitannins [28] as well as of fatty acids such as punicic acid (PA, 9c, 11t, 13c−linolenic acid). Epidemiological studies indicated that the consumption of foods rich in specific polyphenols and anthocyanins are associated with lower incidence of chronic inflammatory diseases due to the proven antioxidant and anti-inflammatory effects of these compounds in humans [29,30,31]. In this context, the anthocyanins intake is associated to an improved HDL antioxidant capacity, as well as enhanced lipoprotein lipase (LPL), and pancreatic lipase activities [32,33], likely related with the beneficial effects of pomegranate on the lipid profile observed in our study. Importantly, a regulation of cholesteryl ester transfer protein (CETP) and lecithin:cholesterol acyltransferase (LCAT) activities by the components of pomegranate should not be discarded since HDL-C and triglycerides were favorably modified.

Accordingly, Hosseini et al. [34] performed a 30-day supplementation with one gram of pomegranate extract (whole fruit with 40% ellagic acid) in patients with obesity, observing a decrease in the levels of cholesterol and triglycerides, and an increase in the concentration of HDL-C compared to the placebo group. Taheri Rouhi et al. [35] administered a supplementation consisting of 1 mL of fresh pomegranate juice or 100 mg of pomegranate seed powder dissolved in 1 mL of water for 21 days in a model of diabetic rats. After the intervention, the group treated with fresh pomegranate juice showed a reduction in the plasma levels of cholesterol, triglycerides, and LDL-C, in addition to an increase in the concentration of HDL-C. Similarly, PA showed hypolipidemic properties related to decreased synthesis and secretion of apo B−100 and triglycerides in liver cells, probably mediated by a reduction in the fatty acid synthase activity and improvement in the activity of enzymes related to β-oxidation [36,37,38]. Therefore, it is likely that some bioactive components of pomegranate may be incorporated into HDL, enhancing their functionality [39].

The other mechanism of action of pomegranate compounds is through activation of peroxisome proliferator-activated receptor alpha (PPARα) and PPAR gamma (PPARγ) [40,41,42]. The PPARs are members of the nuclear receptor superfamily, which are implicated in the regulation of the expression of genes related to HDL metabolism as overexpression of LPL [43], suggesting a PPAR-dependent remodeling of HDL structure. In this context, our results showed that the lipid composition of HDL particles was modified after supplementation with MiPo, independently of high triglycerides concentrations. We also observed a significant decrease in the triglycerides and increases in the cholesterol and phospholipids contents in HDL particles. Consequently, only the HDL-Tg/Pho ratio decreased, whereas the HDL-C/Pho and HDL-SM/Pho ratios did not change. These results suggested that the numbers of HDL particles increased and that these particles were triglycerides poor after intervention with MiPo. However, the lipid modification of HDL was not reflected by changes on the size distribution. Moreover, no particular subclass of HDL was affected by the triglyceride decrease; we observed that all subclasses had a lower content of triglycerides. The content of triglycerides seems to affect the capacity of HDL to accept cholesterol from tissue [44,45,46,47]; impaired cholesterol transport, characteristic of patients with metabolic syndrome [45], has been associated with HDL particles enriched with triglycerides [46] and saturated fatty acids [47].

Therefore, the biological activities and atheroprotective functions of HDL are linked to the particle structure. Our results showed that HDL from rabbits supplemented with MiPo increased PON1 activity and accordingly became more resistant to oxidation, as determined by the kinetics of Cu^2+^-catalyzed conjugated dienes formation within HDL; such HDL modifications were concurrent with an improvement of the endothelial function compared with the placebo group. PON1 is an enzyme carried in plasma by HDL that confers most of the antioxidant properties of these lipoproteins [8,42]. Estrada et al. [20] demonstrated that both *PON1* gene expression and PON1 activity increased in mice fed a high-fat diet after five months of oral supplementation with pomegranate juice. Rock et al. [48] observed an increase in PON1 activity in diabetic patients after supplementation during five weeks with pomegranate juice and polyphenol extract (95% ellagitannic and 5% ellagic acid). Hence, the lipid changes in HDL, particularly reflected by a decreased proportion of triglycerides, could be associated to the increased PON1 activity and *t*_lag_ of conjugated dienes formation after MiPo supplementation. Different studies described small HDL3c subclass rich in phospholipids carrying more PON1 than large Pho-poor-HDL particles, probably due to a more favorable surface tension or PON1 desorption from hepatocyte membranes by HDL [49]. The other explanation is a possible enhancement of the intestinal HDL synthesis induced by the different bioactive components of pomegranate, likely through the activation of PPARs [50].

The co-incubation of HDL isolated from plasma in fasting conditions, after supplementation with MiPo, induced an improvement in endothelial function using a model of aorta rings. Accordingly, Estrada et al. [21] showed that MiPo supplementation after 30 days reversed the negative effects on endothelial function of HDL generated during the postprandial period in women with ACS. Therefore, the modifications of HDL structure and the enhancement of their antioxidant properties may explain the beneficial effects vis-à-vis the endothelial function [51]. Whether some bioactive compounds of pomegranate were associated to HDL structure [52] and delivered to the cells via these lipoproteins [11] remains to be elucidated.

Of particular interest, our results corroborate that the beneficial properties on lipid profile of the fresh pomegranate are conserved by the MiPo [20,34]. The microencapsulation has important advantages for functional foods; seasonal and endemic fruits, as pomegranate, may be efficiently distributed and consumed along the year. Among the different techniques for microencapsulation, such as coacervation-phase separation, pan-coating process, solvent evaporation, air suspension, interfacial polymerization, and multi-orifice centrifugal process, spray-drying is the most common method for liquids containing phenolic compounds [53]. Moreover, the use of natural polymers (maltodextrin and Arabic gum) as coating material increases the stability and oxidative protection of functional ingredients. Hence, the microencapsulation is a useful to protect the bioactive ingredients of pomegranate as observed in this study [54].

## 4. Materials and Methods

### 4.1. Animals

This study was a randomized, placebo-controlled trial, that aimed to determine the effect of microencapsulated pomegranate (MiPo).

Male New Zealand white rabbits weighing 2.0–2.5 kg were randomized into two different groups: one group (*n* = 6) received 1g of MiPo supplementation (equivalent in polyphenol content to approximately 250 mL of fresh pomegranate juice) daily for 30 days (MiPo group). The second group (*n* = 6) received 1 g/day of maltodextrin (encapsulating agent) during the same period of 30 days (placebo group). Both groups had free access to a normal chow diet providing 23.6%, 8.7%, and 67.6% of the total calories as protein, fat, and carbohydrates, respectively (Laboratory rabbit diet No. 5321; LabDiet, St. Louis, MO, USA), and water ad libitum. Rabbits were maintained under a 12/12 h light/dark cycle.

All procedures were performed in accordance with the Guide for the Care and Use of Laboratory Animals [55]. The protocol was approved by both the Scientific and the Institutional Committee for the Care and Use of Laboratory Animal (CICUAL: 18-1049) from the Instituto Nacional de Cardiología Ignacio Chávez.

### 4.2. Microencapsulated Pomegranate (MiPo)

The MiPo was prepared as previously described by Estrada et al. [21]. A previous study reported that the MiPo has the same beneficial effects on lipid profile and PON1 activity as the fresh juice [20]. Briefly, fresh pomegranate seeds and sarcotestas were ground and sieved. The resulting liquid was dried and ground until a fine powder was obtained, followed by an extraction with ethanol and water (1:1, *v*/*v*). The extract was mixed with maltodextrin and dextrose at a 4:1 (*v*/*v*) ratio (Amfher Foods, S.A. de C.V., Mexico City, Mexico) and Arabic gum (Sigma-Aldrich Chemical Co., St. Louis, MO, USA) as coating materials. The microencapsulated powder showed excellent stability at room temperature and stored in darkness.

### 4.3. Blood Samples

After 12 h overnight fasting, blood samples were drawn from the central artery of the ear in tubes with sodium heparin (15 IU/mL). Blood samples were centrifuged for 15 min at 2500 rpm after collection. The pre- and post-supplementation plasma samples were separated into 500 µL aliquots and immediately frozen at −70 °C. For HDL isolation, pre- and post-supplementation plasma samples were simultaneously thawed, immediately processed as described below, and HDL structure and functionality were analyzed within the following 24 h.

### 4.4. Biochemical Analyses

The total cholesterol, triglycerides and glucose plasma concentrations were determined by enzymatic colorimetric methods (Randox Laboratories Ltd., Crumlin, Antrim, U.K.). Sphingomyelin (SM) concentrations were assessed by a commercial colorimetric test (Cayman Chemical, Ann Arbor, MI, USA). The phosphotungstic acid−Mg^2+^ method (Randox Laboratories Ltd., Crumlin, Antrim, U.K.) was used to precipitate the apo B-containing lipoproteins (i.e., Very Low-Density Lipoproteins/Low-Density Lipoprotein); then HDL-C and HDL-Tg plasma concentrations were determined in the supernatant fraction by the same enzymatic colorimetric methods (Randox Laboratories Ltd., Crumlin, Antrim, U.K.), whereas HDL-phospholipids (HDL-Pho) were determined with a commercial kit (Wako Chemicals, Richmond, VA, USA).

HDL-SM and non−HDL-SM were determined by analytical ultracentrifugation. Plasma samples were adjusted at a density of 1.063 g/mL with solid KBr. The bottom (HDL) and top (non-HDL lipoproteins) of the tubes after ultracentrifugation were quantitatively recovered and analyzed using the same colorimetric assay kit mentioned above (Cayman Chemical, Ann Arbor, MI, USA). All the determinations were performed following the manufacturer’s instructions.

### 4.5. HDL Subclasses Composition Assessment

HDL were isolated by ultracentrifugation using density solution (1.21 g/mL) as previously described [56]. Recovered HDL was dialyzed against 0.09 M Tris/0.08 M boric acid/3 mM ethylenediaminetetraacetic acid (EDTA) buffer, pH 8.4. To further separate the recovered fractions by their hydrodynamic diameter, 25 µg of HDL protein were run in a nondenaturing 3–30% gradient polyacrylamide gel electrophoresis. The gels were stained for cholesterol, triglycerides, phospholipids, and sphingomyelin using in-house prepared enzymatic mixtures [26,57]. The gels were incubated for 30 min at 37 °C in darkness, washed with water, and were scanned to obtain an image (lipid image) in a GS-670 densitometer (Bio-Rad laboratories, Hercules, CA, USA). Next, gels were re-stained with Coomassie blue R-250 in a GS-800 Calibrated Densitometer (BioRad Laboratories, Hercules, CA, USA) to detect proteins. To delimit HDL subclasses (lipids and protein), an optical densitometry protein scan was analyzed using globular proteins as the diameter reference (thyroglobulin, 17 nm; ferritin, 12.2 nm; catalase, 10.4 nm; lactate dehydrogenase, 8.2 nm; and albumin, 7.1 nm; high-molecular weight calibration kit; Amersham Pharmacia Biotech, Buckinghamshire, U.K.). Each subclass was measured using VisionWorks^®^ Life Science Software version 8.20 (Ultra-Violet Products Ltd., Nuffield, Cambridge, U.K.) and is expressed as the percentage of total HDL protein and lipid area of the densitogram (7.94–13.59 nm) [6,26,56].

### 4.6. Kinetics of HDL Conjugated Dienes Formation (Oxidation)

The primary products of lipid peroxidation are lipid hydroperoxides and can directly be measured. The kinetics of copper ion-induced formation of conjugated dienes was determined as described previously [58] with modifications. Briefly, isolated samples of HDL were adjusted to 75 µg/mL of protein in 10 mM phosphate buffer (PBS), pH = 7.4. The oxidation of HDL was initiated by addition of freshly prepared 10 µM CuSO_4_ solution. The kinetic of conjugated dienes formation was continuously monitored by measuring the increase of the absorbance at 234 nm, every 2 min for 180 min, at 37 °C. The lag time (*t*_lag_) was determined by the intersection of the linear regressions of the lag and propagation (log) phases (Figure S1). The *t*_lag_ was considered as the time from the addition of the Cu^2+^ until the start of the log phase of diene formation; the longer the *t*_lag_ the higher the resistance to oxidation of the HDL.

### 4.7. Paraoxonase-1 (PON1) Activity

PON1 was determined using phenylacetate as a substrate [59]. The assay mixture included 1 mM phenylacetate and 0.9 mM CaCl_2_ in 20 mM Tris−HCl, pH 8, and 10 μL serum (diluted 1:40). The rate of hydrolysis was measured spectrophotometrically at 270 nm (UV−VIS Beckman Coulter, Brea, CA, USA). The absorbance at 270 nm for the reaction was 1310 M^−1^cm^−1^. The enzyme activity is expressed as the number of micromoles of phenylacetate hydrolyzed per minute, per milliliter of serum.

### 4.8. Vascular Reactivity of Aorta Rings

The effect of HDL on endothelial function was evaluated using aorta rings of New Zealand rabbits in an isolated organ bath as previously described [60]. Aorta rings were incubated with HDL to a final concentration of 50 mg/dL of cholesterol in Krebs solution (118.1 mM NaCl, 4.7 mM KCl, 1.2 mM MgSO_4_, 1.20 mM KH_2_PO_4_, 2.5 mM CaCl_2_, 25.0 mM NaHCO_3_, and 11.1 mM glucose at pH 7.4), continuously bubbled with 95% O_2_ and 5% CO_2_, at 37 °C, 1 h prior to the test. After an equilibrium period, aorta rings were precontracted with 3 × 10^−4^ M phenylephrine. The endothelium-mediated relaxation (vasodilation) was evaluated using a dose–response curve to acetylcholine (5 × 10^−9^–8 × 10^−7^ M). Contractions were measured isometrically with an FT−03 Grass Force Displacement Transducer and recorded on a Grass Polygraph (Model 7D, Grass Medical Instruments, Quincy, MA, USA). All experiments were run in duplicate, and vasodilation of aorta rings was expressed as the percentage of pre-contraction with phenylephrine.

### 4.9. Statistical Analysis

Data are presented as median and interquartile range. Wilcoxon’s paired test or the Mann–Whitney U test were performed to compare the pre- and post-supplementation or placebo and MiPo groups, respectively. The normal distribution of the vasodilation percentages was verified by the Kolmogorov–Smirnov test; since these data were normally distributed, data are presented as media ± standard error and an ANOVA test and least significant difference (LSD) post hoc analysis were performed to compare the pre- and post-supplementation results obtained with each acetylcholine concentration. Statistical significance was considered at *p* < 0.05. All statistics were performed with SPSS 24.0 software (SPSS Inc. IBM, Chicago, IL, USA).

## 5. Conclusions

In conclusion, MiPo had beneficial effects on triglycerides, total cholesterol, LDL-C, HDL-C, HDL-Tg plasma levels, increased resistance to HDL oxidation and PON1 activity in rabbits. In addition, endothelial-dependent vasodilation after MiPo supplementation was significantly better in the presence of isolated HDL after MiPo supplementation than that in the presence of HDL from plasma in basal conditions. All these beneficial effects were demonstrated in an animal model that is characterized by mild triglycerides increases during the postprandial state, strongly suggesting that pomegranate enhances HDL functionality, independently of the its effects on triglycerides plasma levels.

## Figures and Tables

**Table 1:** Blood glucose levels, weight, lipid profile, and sphingomyelin before and after intervention with microencapsulated pomegranate (MiPo) or placebo group.

Parameter		Placebo *n* = 6	MiPo *n* = 6
Weight (kg)	Baseline After 30 days	2.2 (2.0–2.3) ^a^ 3.1 (2.9–3.2)	2.1 (2.0–2.2) ^b^ 3.0 (2.9–3.0)
Glucose (mg/dL)	Baseline After 30 days	76.6 (66.9–84.5) 80.9 (75.1–90.1)	85.0 (76.8–99.4) 79.9 (73.5–83.2)
Total cholesterol (mg/dL)	Baseline After 30 days	52.5 (45.2–59.2) 54.7 (47.4–62.4)	52.3 (45.3–71.7) ^b^ 41.4 (33.9–54.8)
Triglycerides (mg/dL)	Baseline After 30 days	75.8 (60.3–85.1) ^a^ 85.9 (68.6–94.4)	65.9 (59.2–85.8)^b^ 50.5 (46.2–56.8) ^c^
Non-HDL-Cholesterol (mg/dL)	Baseline After 30 days	20.9 (16.5–28.9) 27.8 (21.3–32.0)	29.2 (17.7–40.6) ^b^ 9.4 (4.3–15.9)^c^
Sphingomyelin (mg/dL)	Baseline After 30 days	5.6 (4.8–7.1) 7.7 (4.2–8.9)	4.6 (3.9–6.5) 5.8 (3.9–7.6)
Non-HDL-SM (mg/dL)	Baseline fter 30 days	3.8(3.3–4.5) 4.0 (2.7–4.8)	3.6 (3.2–4.4) ^b^ 2.6 (2.0–3.0)^c^
HDL-C (mg/dL)	Baseline After 30 days	30.4 (27.2–33.6) ^a^ 27.2 (24.8–29.2)	32.6 (30.6–34.6) ^b^ 37.6 (34.0–40.3) ^c^
HDL-Tg (mg/dL)	Baseline After 30 days	45.2 (23.6–55.4) 40.8 (31.7–46.2)	41.1 (33–49.6) ^b^ 30.2 (23.5–33.6)
HDL-Pho (mg/dL)	Baseline After 30 days	52.0 (44.4–61.0) 46.1 (42.1–52.3)	60.4 (56.1–64.0) ^b^ 64.8 (59.3–68.0)
HDL-SM (mg/dL)	Baseline After 30 days	1.6 (1.2–1.9) ^a^ 2.5 (1.5–4.2)	1.5 (1.1–2.2) 1.4 (0.9–2.1)

Note: HDL-C: HDL–cholesterol, HDL-Tg: HDL-triglycerides, HDL-Pho: HDL-phospholipids, HDL-SM: HDL-sphingomyelin. Data are expressed as median (interquartile range). Mann–Whitney U test for non-normal distribution. ^a^ *p* < 0.05, vs. after 30 days of administration with the encapsulating agent of MiPo, maltodextrin (Placebo). ^b^ *p* < 0.05, vs. after 30 days of supplementation with MiPo. ^c^ *p* < 0.05, placebo vs. MiPo after 30 days.

**Table 2:** Lipid composition of high-density lipoproteins (HDL) subclasses.

HDL Lipids Subclass		Placebo *n* = 6	MiPo *n* = 6
**Cholesterol (mg/dL)**			
HDL 2b	Baseline After 30 days	12.1 (8.7–15.6) 11.7 (6.8–17.8)	11.8 (6.9–16.7) 13.9 (10.0–18.0)
HDL 2a	Baseline After 30 days	4.7 (3.4–6.0) ^a^ 3.8 (2.7–5.2)	4.6 (3.9–5.2) ^a^ 4.6 (3.9–5.3) ^c^
HDL 3a	Baseline After 30 days	6.7 (5.5–7.9) 5.7 (4.9–6.6)	8.3 (6.1–10.6) ^b^ 7.6 (6.0–9.0)
HDL 3b	Baseline After 30 days	2.8 (2.1–3.5) 2.5 (1.3–4.7)	3.6 (2.3–4.9) 4.1 (2.4–5.8)
HDL 3c	Baseline After 30 days	3.5 (1.1–5.9) 3.4 (1.1–5.8)	4.0 (1.4–6.5) ^b^ 6.9 (3.2–9.7)^c^
**Triglycerides (mg/dL)**			
HDL 2b	Baseline After 30 days	17.5 (4.9–30.1) 16.7 (9.6–23.9)	16.6 (8.0–25.3) 12.4 (6.7–18.1)^c^
HDL 2a	Baseline After 30 days	6.1 (2.2–10.1) 5.6 (3.1–8.1)	5.7 (3.3–8.4) ^b^ 4.1 (2.1–6.1)
HDL 3a	Baseline After 30 days	9.0 (2.9–15.1) 8.6 (7.0–9.4)	9.6 (4.8–14.4) ^b^ 6.7 (4.1–9.2)
HDL 3b	Baseline After 30 days	4.3 (2.9–7.6) 3.9 (2.6–4.7)	5.0 (2.1–7.9) ^b^ 2.8 (1.8–3.9)
HDL 3c	Baseline After 30 days	4.5 (3.1–6.3) 4.3 (2.3–7.0)	4.9 (1.1–8.7) ^b^ 3.1 (0.5–6.6)
**Phospholipids (mg/dL)**			
HDL 2b	Baseline After 30 days	22.6 (10.2–35.8) 19.5 (11.7–31.4)	24.6 (15.8–33.3) 27.5 (23.1–31.8)
HDL 2a	Baseline After 30 days	8.2 (6.3–10.1) 6.3 (4.8–8.1)	8.5 (6.4–10.7) 9.8 (7.6–12.0)
HDL 3a	Baseline After 30 days	11.8 (8.7–14.8) 12.2 (8.2–16.3)	15.6 (8.2–23.0) 14.3 (9.5–19.2)
HDL 3b	Baseline After 30 days	5.1 (1.5–8.6) 6.1 (2.1–9.5)	6.7 (2.1–11.3) 6.9 (2.9–10.9)
HDL 3c	Baseline After 30 days	4.9 (1.9–10.7) 2.9 (1.2–3.9)	4.1 (1.2–8.4) 4.7 (2.1–7.4)
**Sphingomyelin (mg/dL)**			
HDL 2b	Baseline After 30 days	0.7 (0.3–1.7) 1.3 (0.8–2.6)	0.8 (0.6–1.6) 0.6 (0.2–0.9)
HDL 2a	Baseline After 30 days	0.3 (0.2–0.4) 0.4 (0.2–0.5)	0.3 (0.1–0.5) 0.2 (0.1–0.5)
HDL 3a	Baseline After 30 days	0.4 (0.2–0.6) 0.5 (0.4–0.7)	0.4 (0.2–0.7) 0.4 (0.3–.0.9)
HDL 3b	Baseline After 30 days	0.1(0.1–0.2) 0.2 (0.0–0.4)	0.2 (0.0–0.3) 0.2 (0.0–0.4)
HDL 3c	Baseline After 30 days	0.2 (0.1–0.2) 0.2 (0.0–0.3)	0.1 (0.1–0.2) 0.1 (0.1–0.2)

Note: Data are expressed as median (interquartile range). Mann–Whitney U test. ^a^ *p* < 0.05 vs. after 30 days of administration with the encapsulating agent of MiPo, maltodextrin (Placebo). ^b^ *p* < 0.05 vs. after 30 days of supplementation with MiPo, ^c^ *p* < 0.05, vs. after 30 days of supplementation with placebo.

## Supplementary Materials

The following are available online at https://www.mdpi.com/1420-3049/25/14/3297/s1, Table S1: Distribution relative HDL size; Figure S1: Method to determine the lag time (*t*_lag_) on the basis of the kinetics of copper-mediated HDL oxidation.
