# Supplementary material for: Microencapsulated Pomegranate Modifies the Composition and Function of High-Density Lipoproteins (HDL) in New Zealand Rabbits
Source: Molecules. 2020 Jul 21;25(14):3297. doi: 10.3390/molecules25143297 (PMC7397439; doi:10.3390/molecules25143297)
Supplement: Supplementary file 1 [file molecules-25-03297-s001.pdf]

Supplementary Materials:

Table S1. Relative HDL size distribution

**HDL subclass (% protein)**		**Placebo** *n* = 6	**MiPo** *n* = 6
**HDL 2b**	Baseline	45.3 (35.1–55.5)	40.2 (29.3–51.1)
	After 30 days	38.9 (27.5–50.3)	35.8 (25.2–46.4)
**HDL 2a**	Baseline	17.8 (15.1–20.5)	16.9 (14.5–19.4)
	After 30 days	19.2 (14.8–23.6)	16.13 (12.3–19.9)
**HDL 3a**	Baseline	24.4 (18.6–30.2)	28.4 (20.6–36.3)
	After 30 days	28.9 (23.5–34.5)	30.8 (26.4–35.3)
**HDL 3b**	Baseline	7.6 (3.7–11.4)	10.3 (6.6–13.9)
	After 30 days	9.1 (3.6–14.6)	12.5 (6.5–17.9)
**HDL 3c**	Baseline	4.9 (1.6–8.3)	4.1 (2.4–5.9)
	After 30 days	3.9 (1.2–7.5)	4.9 (1.0–10.4)

Note: HDL: high-density lipoproteins, MiPo: microencapsulated pomegranate. Data are expressed as median (interquartile range), before and after of 30 days of supplementation with MiPo or administration with maltodextrin as placebo. Mann-Whitney U test for non-normal distribution

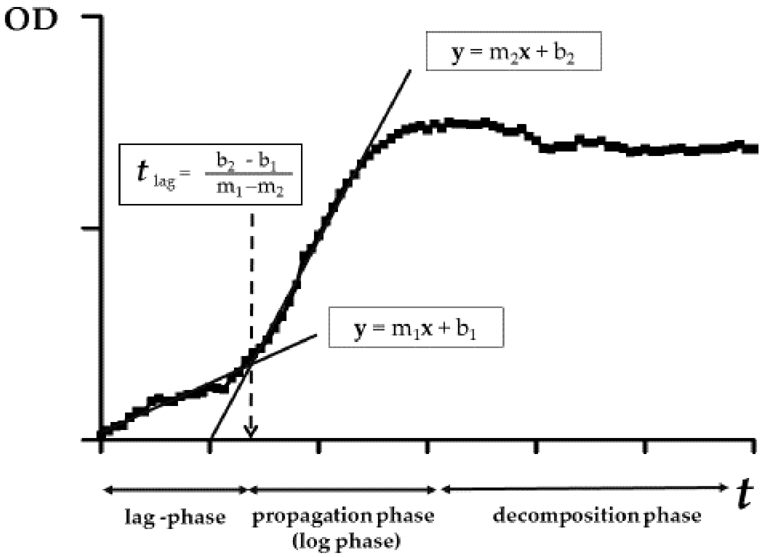

Figure S1. Method to determine the lag time (t_{lag}) on the basis of the kinetics of copper-mediated HDL oxidation. The temporal changes in absorbance at 234 nm is divided into three phases, a lag phase, a propagation phase (log phase) and a decomposition phase. Both the lag and lineal phases were quantified by linear regression of the lag and log phases of the curve. The x value of the common point to the line equations ($y=m_1x+b_1$ and $y=m_2x+b_2$) is considered the t_{lag}.
